# Localized Utilization of Silica Fume in High-Volume Fly Ash Concrete Through Densification of the Interfacial Transition Zone

**DOI:** 10.3390/ma19184009

**Published:** 2026-09-20

**Authors:** Ciren Wangdui, Yu Chen, Erlin Wu, Soufian El Mghari, Yongquan Zhang, Xiaodong Wang, Sen Du

**Affiliations:** 1 Xizang Communication Surveying and Design Institute Co., Ltd., Lhasa 850000, China; 2 School of Civil Engineering and Mechanics, Yanshan University, Qinhuangdao 066004, China; 3 Transportation Institute, Inner Mongolia University, Hohhot 010021, China

**Keywords:** interfacial transition zone, silica fume, high-volume fly ash, pretreatment, optimization

## Abstract

Interfacial transition zone (ITZ) is usually the weakest area in concrete, especially in high-volume fly ash (HVFA) concrete, where most fly ash particles remain unreacted due to their relatively low reactivity. In this case, some supplementary cementitious materials featuring high reactivity, such as silica fume, are usually added directly into HVFA concrete to improve its ITZ. This study proposed a targeted aggregate pretreatment method, which only employed silica fume in the ITZ to enhance its material-use advantages. The approach involved pre-coating coarse aggregate with a silica fume-modified cement paste layer and casting HVFA concrete using such pretreated coarse aggregate. The influence of five key parameters during the preparation of silica fume-modified paste, namely the water-to-cementitious material ratio, the content of silica fume, the mass ratio of coarse aggregate to cement paste, the mixing time for blending coarse aggregate and paste, and the drying time after mixing, on the void fraction of the ITZ of HVFA concrete cast with such coated coarse aggregate was investigated. Response surface optimization identified W/CM = 0.3, SF/CM = 9%, Ca/CP = 4, and a mixing time of 3 min as the factor settings for minimizing the ITZ void fraction within the investigated domain. A drying time of 5 h was selected for experimental confirmation, yielding an experimentally measured ITZ void fraction of only 21.4%. Furthermore, compared to the control groups, the optimized HVFA concrete exhibited improved compressive strength and water absorption performance and densified ITZ microstructure. Notably, the silica fume consumption in this approach was 2.8–3.5 times lower than the commonly reported dosage range of 8–10%, demonstrating material-use advantages alongside considerable performance enhancement.

## 1. Introduction

Concrete, as a multiphase composite material, generally comprises cement paste, aggregate, and the interface between them. Interfacial transition zone (ITZ) between the cement paste and aggregate is widely regarded as the weakest area in concrete due to the presence of high porosity and oriented growth of portlandite (CH) crystals in this region [1,2]. The formation of ITZ is primarily attributed to the wall effect that occurs during the placement of fresh concrete, when the significant difference in size between aggregate particles and cement grains leads to a locally higher water-to-cement ratio around the aggregate [3,4]. As a result, due to the higher CH content and loose microstructure within the ITZ, its mechanical strength is significantly lower than that of the cement paste, making it prone to microcrack initiation and propagation under external loads. Furthermore, the water absorption of the ITZ can be more than 90 times greater than that of the cement paste [5]. When exposed to sulfate or chloride attack, the ITZ serves as a primary pathway for the rapid ingress of aggressive ions, accelerating the deterioration of concrete. Therefore, enhancing the ITZ has become a critical issue in improving the overall performance of concrete.

In recent years, in order to reduce carbon emissions of the concrete industry, industrial by-products such as fly ash, silica fume, and slag have been increasingly used to partially replace cement in concrete, thereby mitigating the environmental impacts. Currently, high-volume fly ash (HVFA) concrete with cement replacement level exceeding 50% and, in some cases, even up to 70% has been developed [6,7]. Many studies have shown that HVFA concrete exhibits improved resistance to sulfate and chloride attacks, primarily due to its lower permeability and reduced content of CH compared to conventional concrete [8,9]. However, the use of fly ash as a cement substitute at a high replacement level also presents certain drawbacks. For instance, the pozzolanic reaction of fly ash is slow, with its reaction degree reaching only approximately 11–15% in HVFA concrete after 28 days of curing [10,11], resulting in a significant portion of fly ash remaining unreacted as inert filler. Consequently, the ITZ in HVFA concrete tends to be weaker and deteriorates more rapidly than that in conventional concrete [12].

Up to now, many studies have proposed various methods to enhance the ITZ in concrete. One approach involves the incorporation of highly reactive supplementary cementitious materials, such as silica fume, which effectively fills pores and consumes CH through pozzolanic reaction, thereby promoting the formation of additional calcium silicate hydrate (C-S-H) gels [13,14]. In recent years, with advances in nanotechnology, nanomaterials, including graphene oxide [15], carbon nanotubes [16], nano-iron oxide [17], and nano-silica [8], have also been introduced into concrete to improve the ITZ of concrete. These nanomaterials possess similar particle sizes to those of C-S-H gels, enabling them not only to fill pores within the concrete but also to act as nucleation sites for hydration products. As a result, the hydration process can be accelerated, which densifies the microstructure of concrete, including the ITZ. However, it should be noted that these methods primarily enhance the ITZ by improving the overall properties of the bulk cementitious matrix.

In order to enhance the ITZ more directly, some targeted strategies focusing on the pretreatment of aggregates have been proposed. Currently, widely adopted methods include chemical pretreatment techniques [18,19] and biomineralization deposition [20,21,22]. In particular, biomineralization deposition utilizes specific microorganisms (e.g., urease-producing or carbonic anhydrase-producing bacteria) to introduce in situ precipitation of inorganic minerals such as CaCO_3_ on the surface of coarse aggregate, directly improving the performance of ITZ of concrete prepared using such coarse aggregate. Besides these methods, the coating of aggregate using nanomaterials has also been applied, such as immersing coarse aggregate in graphene oxide suspension [23] and nano-silica solution [24]. Through this way, the nanomaterials can be directly applied in the ITZ of concrete, resulting in a densified microstructure. However, in practical application, both accelerated carbonation and biomineralization deposition are limited by the need for complex and costly equipment, while the employment of nanomaterials coating can increase the production cost of concrete due to their high price.

To effectively improve the ITZ in HVFA concrete while meeting low-carbon and economic requirements, this study proposed an efficient aggregate pretreatment approach using silica fume. This approach involved coating coarse aggregate with a layer of silica fume-modified cement paste, which can form a stably attached and highly reactive coating after a period of drying. During the subsequent casting of concrete using such pretreated coarse aggregate, this coating layer can result in the formation of a silica fume-enhanced ITZ. To quantitatively evaluate the microstructural improvement of the ITZ achieved by this approach, backscattered electron (BSE) imaging analysis was conducted to measure the void fraction of the ITZ. Based on response surface methodology, an experimental design was implemented with the objective of optimizing the pretreatment procedures through minimizing ITZ void fraction, during which five key factors were investigated on their effects on the ITZ void fraction. In addition, comparisons of macroscopic properties and microstructural densification between optimized HVFA concrete and control groups were conducted to validate the effectiveness of the proposed approach. At last, the material-use advantages of the employment of silica fume in the ITZ of HVFA concrete were compared with its typically used content.

Although previous research has demonstrated the feasibility of modifying the ITZ through slag-containing cementitious coatings [6], whether the same optimization strategy is applicable to silica fume-containing coating systems remains unclear. Silica fume differs substantially from slag in particle size, reactivity, and its contribution to particle packing and pozzolanic reactions, which may lead to different microstructural evolution and optimum coating conditions. Therefore, the objective of this study is not simply to replace slag with silica fume, but to systematically elucidate the effects of silica fume on the microstructure of the ITZ and establish the corresponding optimum preparation conditions. Particular attention is given to the relationship between the coating parameters and ITZ void fraction, thereby providing a quantitative basis for the targeted utilization of silica fume for ITZ modification in HVFA concrete.

## 2. Experimental Study

### 2.1. Materials

This study employed type P•II 42.5 Portland cement and silica fume to prepare the modified cement paste. Cement was purchased from Asano Co., Ltd. in the city of Qinhuangdao, China, while silica fume was obtained from Sichuan Evande New Materials Co., Ltd., Chengdu, China. For the preparation of HVFA concrete, Class F fly ash and the same type of cement were used. Fly ash was obtained from Huaneng Shang’an Power Plant, Shijiazhuang, China. The chemical compositions of these three cementitious materials are presented in Table 1. The physical properties of cement, silica fume, and fly ash are listed in Table 2.

Coarse aggregate used in this study was crushed limestone with a continuous gradation, a particle size range of 5 to 20 mm, and an apparent density of 2711 kg/m^3^. Fine aggregate consisted of natural river sand with a fineness modulus of 2.7 and a maximum particle size of 4.75 mm. The apparent density of the sand is 2618 kg/m^3^. Both coarse and fine aggregates were treated to achieve saturated surface dry (SSD) condition prior to use. To enhance the workability and performance of the high-volume fly ash concrete, a polycarboxylate ether-based superplasticizer with a solid content of 34.4% was employed. This admixture is essential for reducing the water content in the mix while maintaining the desired consistency.

### 2.2. Pretreatment of Coarse Aggregate

In this study, silica fume-modified cement paste was used to coat the coarse aggregate, aiming to achieve targeted application of silica fume in the ITZ of HVFA concrete. In order to obtain the optimized result, five factors were considered during the pretreatment of coarse aggregate using silica fume-modified cement paste, namely the water-to-cementitious material ratio of the cement paste (W/CM, *X*_1_), the silica fume content in cementitious materials (SF/CM, *X*_2_), the mass ratio of coarse aggregate to cement paste (CA/CP, *X*_3_), the mixing time of coarse aggregate and cement paste (MT, *X*_4_), and the drying time after their mixing (DT, *X*_5_). It should be noted that CP includes all constituents of the coating paste, i.e., cement, silica fume, and mixing water. The value ranges for these factors were set as 0.3–0.4, 0–10%, 1.5–4.5, 2–10 min, and 0–8 h, respectively, as shown in Table 3. A five-factor, five-level central composite design (CCD) based on response surface methodology (RSM) was employed to investigate the effects of the selected experimental factors. In the CCD, 0 represents the center point of the design space, while ±1 and ±α indicate the distances of factorial points and axial points from the center, respectively. The experimental design was generated using Design-Expert (version 13) software, with α set to 1.82. A total of 26 groups were designed, as listed in Table 4. It should be noted that the experimental runs were performed in a randomized order according to the run sequence generated by Design-Expert to minimize potential systematic effects associated with experimental order. Five repeated center-point runs (#3, #4, #6, #11, and #20) were included to provide an estimate of pure experimental error and repeatability and to facilitate assessing the response-surface curvature [25].

In the present study, ITZ void fraction was selected as the sole response variable for response surface optimization because the primary objective of the proposed aggregate pretreatment strategy was to improve the microstructural quality of the aggregate–paste interface in HVFA concrete. The five pretreatment parameters investigated in the response surface design were specifically intended to control the characteristics of the silica fume-modified coating and its subsequent influence on the ITZ. Therefore, ITZ void fraction was considered the most direct and sensitive response for identifying the pretreatment conditions that promoted ITZ densification.

Other properties, including workability, compressive strength, sorptivity, and total porosity, are also important for the overall performance of concrete. However, they reflect the combined effects of the bulk matrix, aggregate, ITZ, and specimen-scale variability and were therefore not used as the primary optimization response for the aggregate pretreatment process. Instead, compressive strength and water transport behavior were evaluated for the optimized mixture and control mixtures to assess whether the microstructural improvement identified through ITZ-based optimization was accompanied by changes in macroscopic concrete performance.

The pretreatment of coarse aggregate was conducted using a laboratory concrete mixer. First, cement, silica fume, and water were placed into the mixer and blended uniformly. Subsequently, coarse aggregate in the SSD condition was added to the silica fume-modified cement paste and mixed for MT minutes. Finally, the cement paste-coated coarse aggregate was removed from the mixer and dried at room temperature for DT hours to obtain the coated coarse aggregate. During this step, moisture loss was intended to occur primarily from the coating layer, while the coarse aggregate was not subjected to additional drying treatment. After the prescribed drying period, the coated coarse aggregate was directly used in the final concrete mixing.

### 2.3. Preparation of HVFA Concrete

Using the pretreated coarse aggregate, HVFA concrete was prepared by mixing it with cement, fly ash, sand, and water at contents of 160, 240, 900, and 120 kg/m^3^, respectively, together with 1000 kg/m^3^ of coarse aggregate. The mixture proportion was reported as mass quantities normalized to the concrete mixture volume of 1 m^3^. The 1000 kg/m^3^ of coarse aggregate represented the mass of the SSD coarse aggregate prior to coating. It should be noted that the cement, silica fume, and water used to prepare the coating paste were additional materials to those used for preparing the concrete. During the preparation of HVFA concrete, the content of fly ash in the total cementitious materials and the water-to-binder ratio were kept at 60% and 0.3, respectively. Note that the fly ash replacement level and water-to-binder ratio refer to the bulk cementitious matrix, rather than to the complete coated-aggregate concrete, including the coating paste. Through this way, the influence of factors on the ITZ microstructure in HVFA concrete can be evaluated while maintaining other variables constant.

HVFA concrete was also prepared using a laboratory concrete mixer. Firstly, cement, fly ash, sand, and pretreated coarse aggregate were mixed in the mixer for 3 min. Subsequently, water was added to the dry materials and mixed for another 3 min. Finally, a superplasticizer dosage of 0.5–0.8% by mass of the cementitious materials was added, followed by an additional 2 min mixing to produce a homogeneous concrete with a slump of 150–180 mm. After mixing, the fresh concrete was poured into cubic molds with 100 mm side length. The surface of the molds was covered with plastic film to prevent moisture evaporation. After curing at room temperature for 24 h, the specimens were demolded and cured under standard conditions (22 ± 2 °C, relative humidity > 98%) until 28 days.

### 2.4. Testing Methods

The microstructure of the ITZ in HVFA concrete was investigated using a TESCAN-VEGA3 scanning electron microscope (SEM) (TESCAN, Brno, Czech Republic). Cubic samples with a size of 1 cm were extracted from the central region of concrete specimens cured for 28 days. The cut samples were immersed in isopropanol for 7 days to terminate hydration. Subsequently, the samples were placed in a vacuum-drying oven and dried for 1 day to effectively remove residual isopropanol and moisture from the interior of the samples. Additionally, a low-viscosity epoxy resin was used to impregnate the samples to ensure the structural integrity of the samples. Finally, the surface of the samples was polished and coated with a conductive layer of gold film. Gold coating was selected based on the available SEM sample preparation and imaging configuration. Because the present BSE analysis focused on relative microstructural contrast and image-based quantification of the void fraction rather than quantitative compositional analysis, the same coating procedure and imaging conditions were applied consistently to all specimens. During the test, the acceleration voltage and beam current of the scanning electron microscope were set to 30 kV and 50 nA, respectively.

For the optimized HVFA concrete and control groups with the size of 100 mm × 100 mm × 100 mm, compressive strength was measured after 28 days of curing using an MTS compression testing machine in accordance with the Chinese standard GB/T 50081-2019 [26]. A loading rate of 0.5 MPa/s was applied during the test. Three replicate specimens were tested to obtain the average compressive strength for each group of HVFA concrete.

For the water sorptivity test, three 50 mm-thick slice specimens were used for each group of HVFA concrete. The slice was cut from the central portion of the cubic concrete (side length of 100 mm) at the curing time of 28 days and then oven-dried at 60 °C for 24 h to remove the moisture within the specimen. Subsequently, the specimen was allowed to cool naturally to room temperature before the water sorptivity test. During the test, the upper and side surfaces of the slice specimen were sealed with plastic film and tape, respectively, ensuring that only the bottom surface was exposed to water. The mass of specimen was recorded at regular time intervals. Finally, the water absorption normalized by the exposed surface area was calculated according to the equation specified in ASTM C1585 [27].

## 3. Results and Discussion

### 3.1. The Void Fraction of ITZ

**Figure 1:**
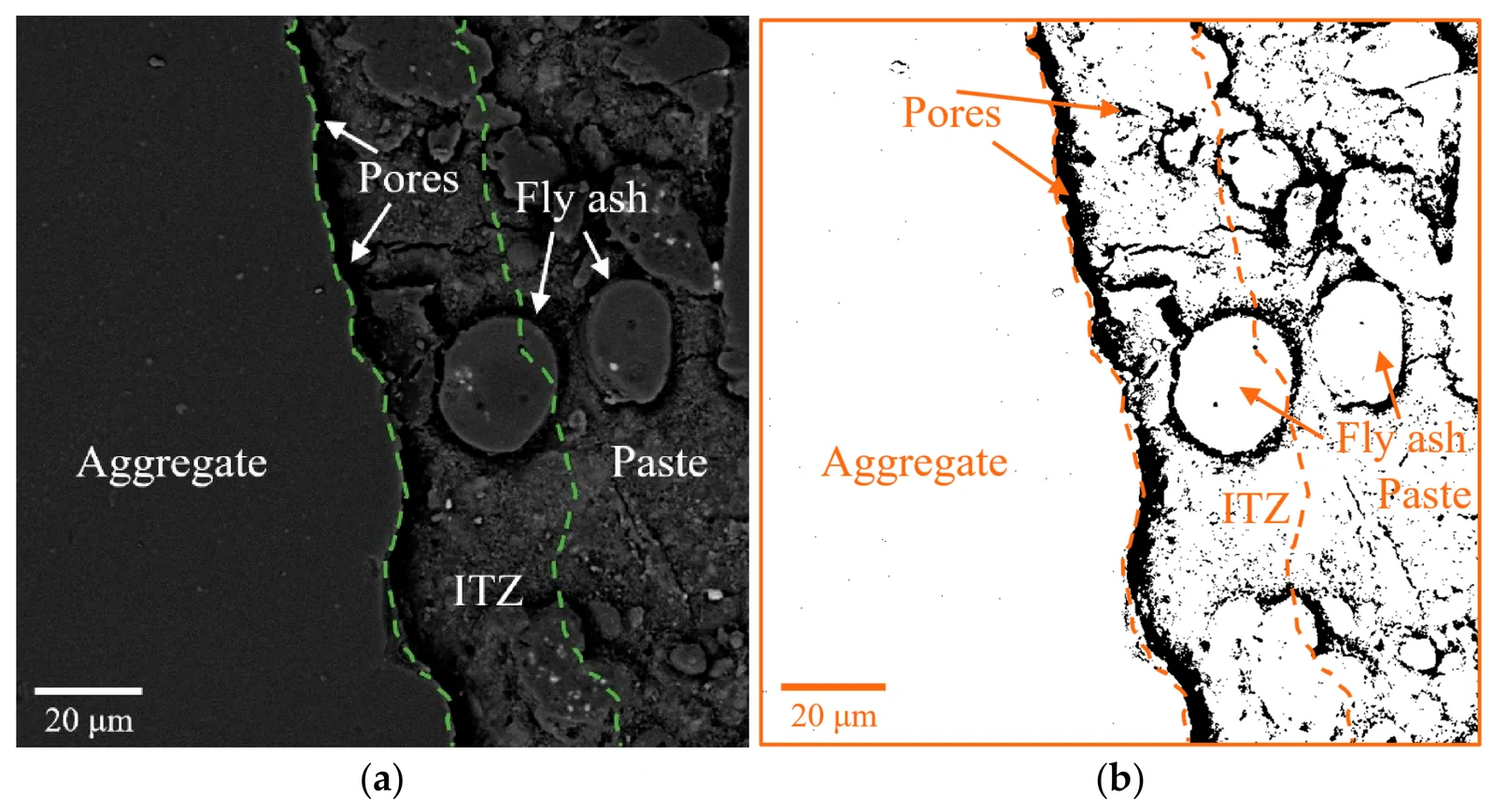
The microstructure of the ITZ in group 18 HVFA concrete (**a**) BSE image (**b**) binary image.

In this study, backscatter electron (BSE) images were employed to calculate the void fraction of the ITZ in HVFA concretes containing different pretreated coarse aggregate. As shown in Figure 1a, taking group #18 as an example, this BSE image reveals the intricate bonding between aggregate and the surrounding paste, with distinct phases identifiable by their gray levels and morphological features. Generally, the average atomic number of a phase determines its gray level in the BSE image [28]. Phases with a higher average atomic number exhibit greater brightness due to the higher number of backscattered electrons they emit, and vice versa. Accordingly, the dark areas at the ITZ in concrete indicate pores and cracks, the bright regions correspond to unhydrated cement particles, and the remaining areas consist of hydration products and unreacted fly ash particles.

In this study, the silica fume-modified coating applied during coarse aggregate pretreatment was conceptually distinguished from the final ITZ formed after concrete mixing and hardening. However, the boundary between the original coating layer and the subsequently developed ITZ could not be reliably distinguished from the BSE images based on grayscale contrast or morphology alone. Therefore, the coating layer was not separately delineated or quantified in the image analysis. Instead, the aggregate surface was first identified manually from the BSE images based on the morphological and grayscale contrast between the coarse aggregate and the surrounding cementitious matrix. The measurement region was then defined as the final ITZ extending from the aggregate surface toward the bulk paste. Its outer boundary was determined based on the observable microstructural transition, specifically at the location beyond which no obvious aggregate-induced gradient in the void structure could be identified, and the paste microstructure became relatively homogeneous. Based on this criterion, the identified ITZ widths were approximately 20–40 μm in this final interfacial region.

In order to quantify the ITZ void fraction (including pores and cracks), the image analysis using ImageJ (version 1.54t, National Institutes of Health, USA) software was conducted by determining the inflection point on the brightness histogram. During this process, separate thresholds were applied to segment voids from the BSE image of different groups of HVFA concrete based on their respective grayscale histograms. Voids in the BSE images were then identified using gray-level thresholding as black pixels [29], as illustrated in the binary image shown in Figure 1b. According to the stereological principle [30], the area fraction observed on a 2D microstructure can be considered equivalent to the volume fraction of the 3D real microstructure, provided the material is randomly and homogeneously distributed. However, the ITZ is a spatially heterogeneous region, and its microstructure may not satisfy the assumptions of random and homogeneous distribution required for a direct equivalence between 2D area fraction and 3D volume fraction. Therefore, in this study, the measured parameter is conservatively described as the 2D BSE-derived void fraction of the ITZ.

In order to obtain statistically valid void fraction results, 50 BSE images were captured per concrete group for quantitative analysis. Specifically, five aggregate–paste interfaces were randomly selected from each specimen, and 10 BSE images were randomly acquired from each interface. Thus, a total of 50 BSE images were analyzed for each mixture group. The 50 images were considered subsamples for characterizing the spatial variability of the ITZ rather than independent experimental replicates. Each image was magnified to 1500 times, corresponding to a field of view measuring 138 μm × 138 μm. The average ITZ porosity results for each concrete group are presented in Table 5. It should be noted that the reported standard deviation (SD) was calculated from the five interface-level mean values.

As shown in Table 5, the void fraction of the ITZ in HVFA concrete ranged from 22% to 39%. Compared to conventional HVFA concrete with similar fly ash content and water-to-binder ratio, a significant reduction in the ITZ void fraction was observed after employing the aggregate treatment. This indicates that the coating layer around coarse aggregates can form the ITZ of HVFA concrete. Notably, due to the higher reactivity of silica fume compared to that of fly ash, the extent of pozzolanic reaction of silica fume was considerably increased in the cement paste [31]. In this study, owing to the high reactivity of silica fume within the ITZ, it participated in pozzolanic reaction with CH, generating C-S-H gel and thereby forming a more densified microstructure.

### 3.2. Model Fitting and Its Validation

Regression analysis was performed on the void fraction of the ITZ to establish the relationship between the five independent variables and the response. In this study, a quadratic polynomial model was selected to present the ITZ void fraction, as shown in Equation (1). It should be emphasized that only variables with a *p*-value less than 0.05 were considered statistically significant and included in the model.

(1) $$\begin{array}{l} Y^{-1.28}=0.0158-0.0047X_{1}+0.0025X_{2}+0.0003X_{3}-0.0003X_{4}-\\ 0.0015X_{1}X_{2}-0.0014X_{1}^{2}-0.0020X_{2}^{2}-0.0034X_{3}^{2}-0.0025X_{4}^{2} \end{array}$$

In order to improve the adequacy of the regression model and stabilize the variance of the response, a Box–Cox transformation was performed using Design-Expert. The Box–Cox analysis recommended a transformation parameter of −1.28 (λ). Therefore, the response was transformed as *Y*^−1.28^, where *Y* represents the measured ITZ void fraction. The regression analysis was subsequently performed using the transformed response. For interpretation and comparison with the experimental results, the predicted values were back-transformed to the original scale of ITZ void fraction before being presented in the subsequent figures and analysis.

The initial response surface model included the linear, two-factor interaction, and quadratic terms corresponding to the five experimental factors. The statistical significance of the individual regression terms was evaluated using a significance level of 0.05. Terms that did not make a statistically significant contribution were considered for removal during model reduction. However, model reduction was conducted while maintaining model hierarchy. In this case, when an interaction or quadratic term was retained, the corresponding lower-order main effects were retained regardless of their individual *p*-values. The resulting reduced model was then used for response surface analysis and optimization. The analysis of variance (ANOVA) results for the terms in Equation (1) and the reduced hierarchical are provided in Table 6.

Moreover, the statistical significance of the model can be evaluated using the model F-value and *p*-value, while the goodness of fit was assessed using the coefficient of determination (R^2^) [32]. The model was statistically significant at the 95% confidence level, with an F-value of 43.85 and a *p*-value of 0.003, as listed in Table 6. The lack-of-fit test yielded an F-value of 0.1073 and a *p*-value of 0.7306. Since the lack-of-fit *p*-value was greater than 0.05, the lack of fit was not statistically significant, indicating that the model adequately represented the experimental data within the investigated design space. The model yielded an R^2^ value of 0.9943, indicating that a high proportion of the variability in the transformed response was explained by the regression model. The adjusted R^2^ value was 0.9717, further demonstrating the adequacy and predictive capability of the model.

**Figure 2:**
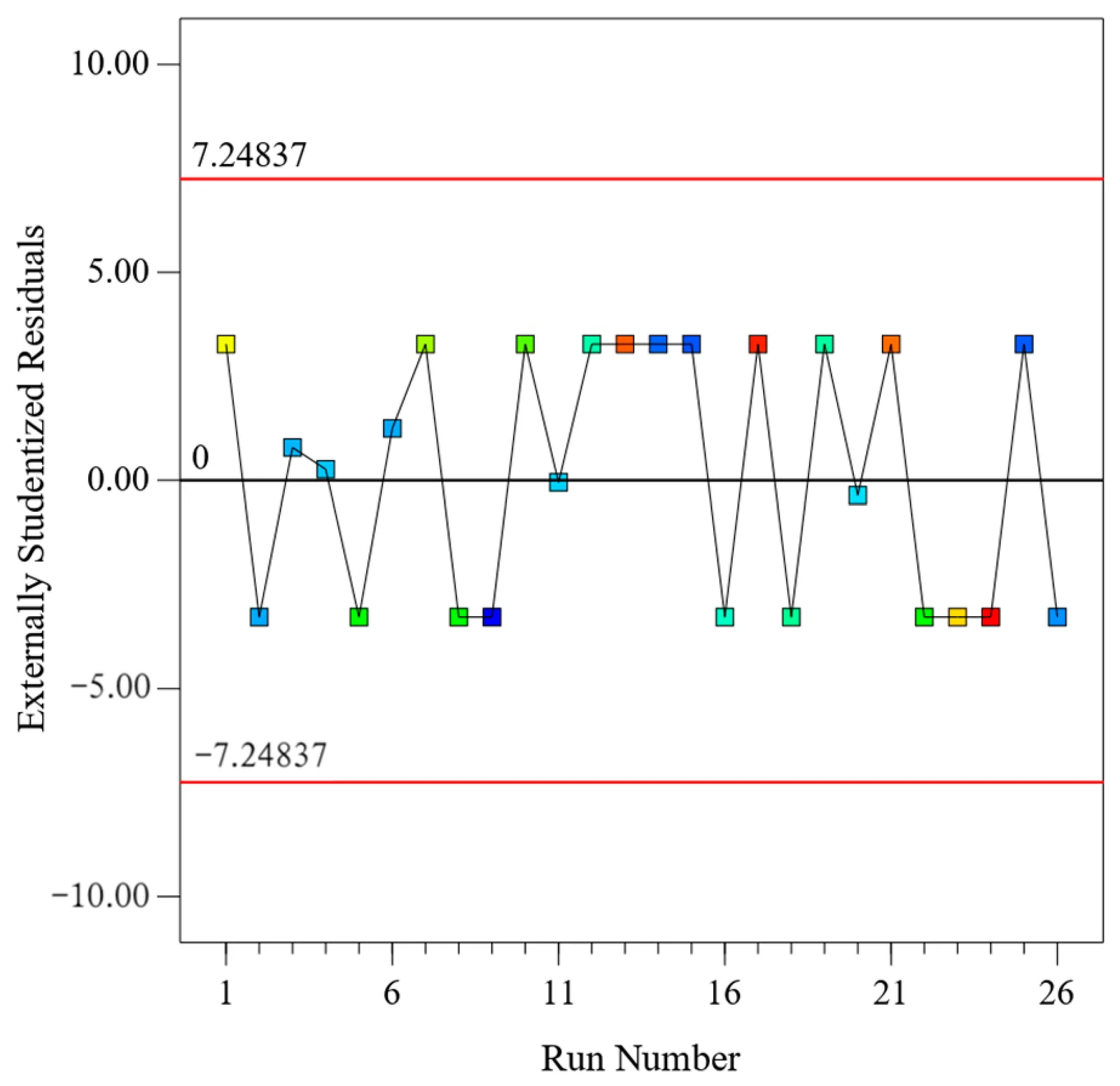
The correlation between residuals and the designed runs of HVFA concrete (colorful squares represent different experimental runs).

**Figure 3:**
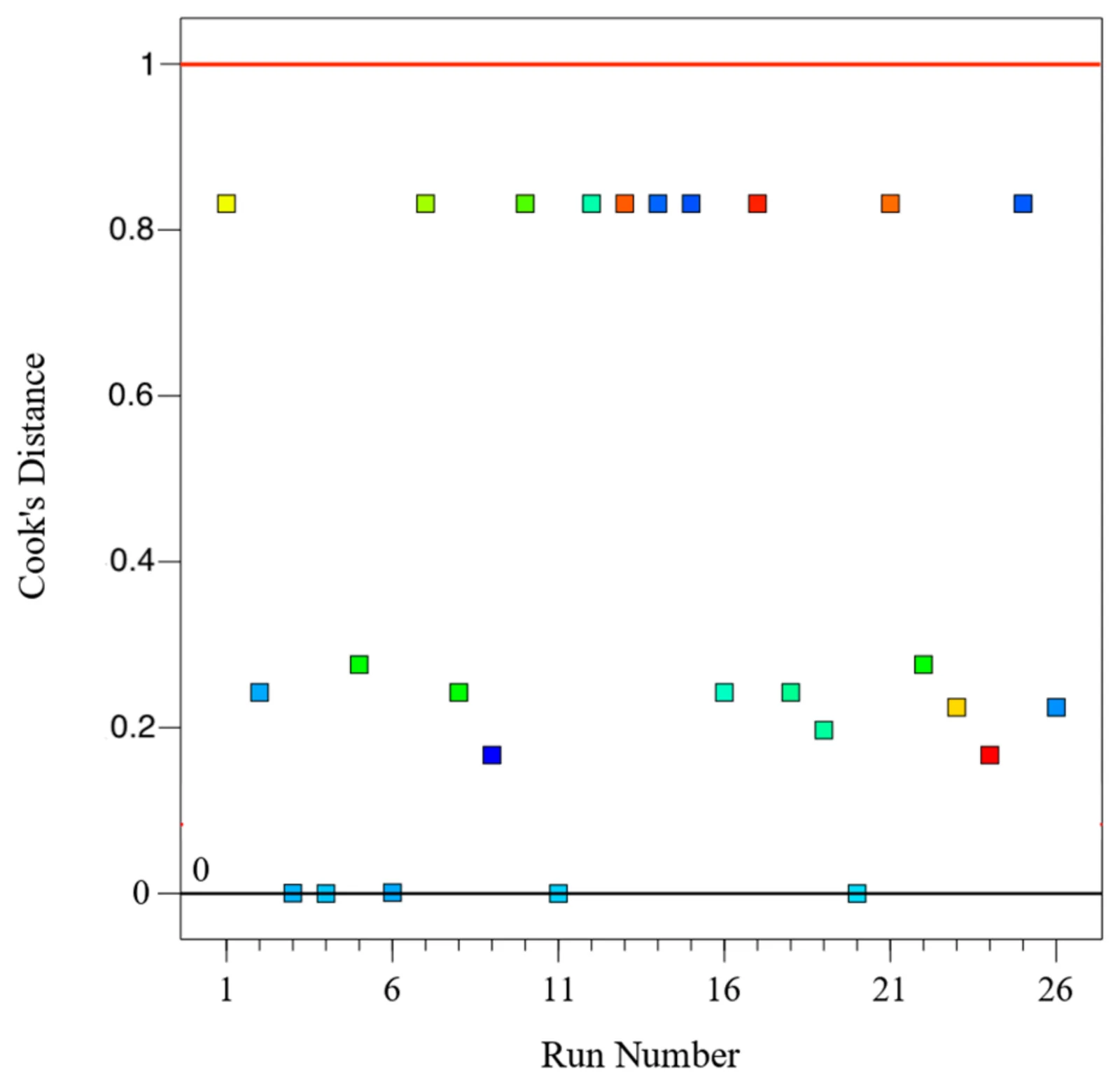
The variation in Cook’s distance for different groups of HVFA concrete (colorful squares represent different experimental runs).

**Figure 4:**
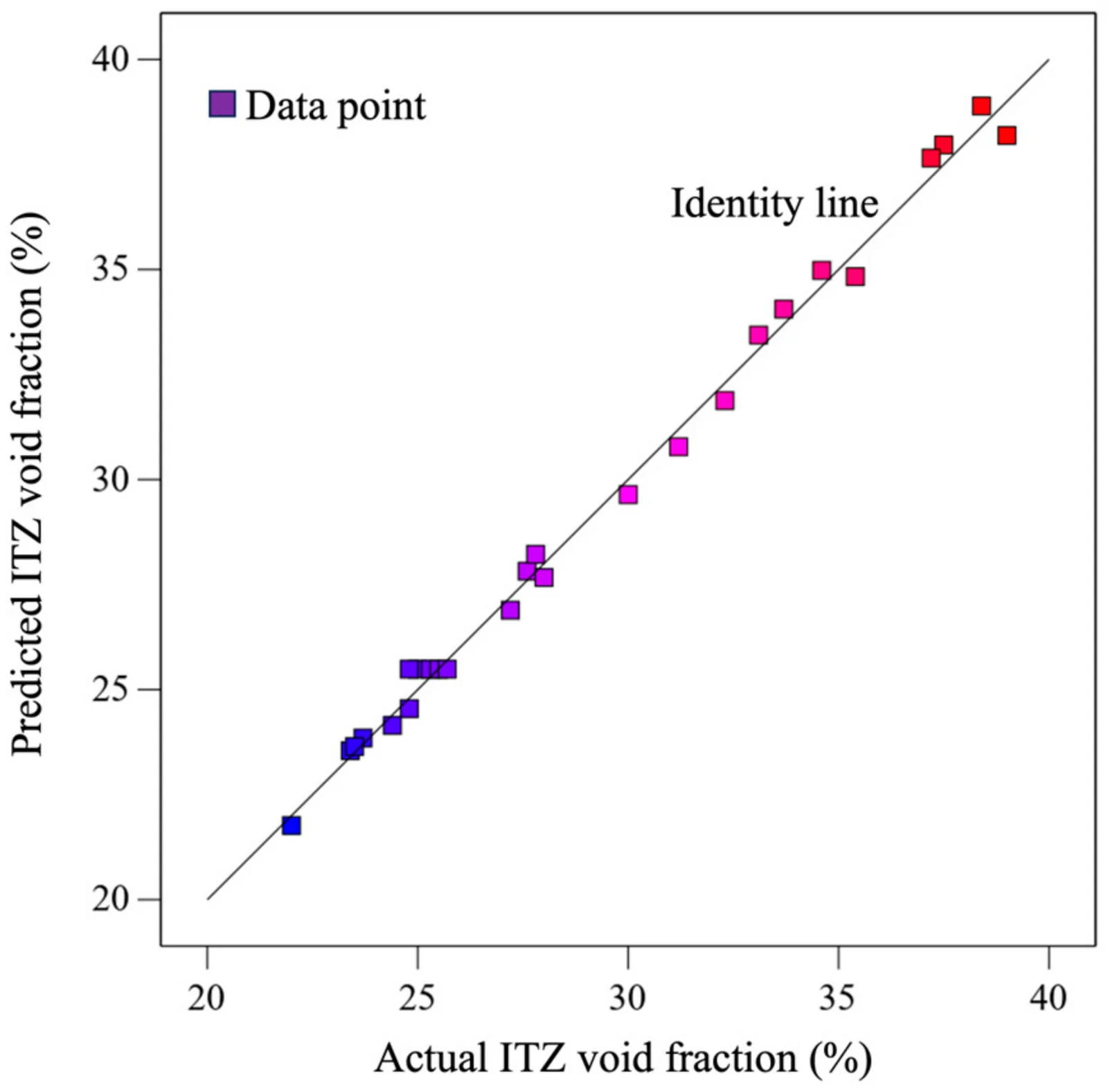
The comparison of the ITZ void fraction between predicted data and actual data (colorful squares represent different experimental runs).

In addition, the residual diagnostic plot showed no obvious abnormalities in the residual distribution or variance (Figure 2), which suggests that the residual diagnostic supported the transformation and model assumptions. Moreover, Figure 3 depicts the Cook’s distance for all mixtures, a regression diagnostic to identify influential observations. A large Cook’s distance indicates a data point has a significant influence on the fitted model and should therefore be investigated as a potential outlier. It can be seen from this figure that the Cook’s distances of all the data points were lower than the conventional threshold of 1, suggesting an absence of outliers for this model. At last, the predicted ITZ void fractions were in good agreement with the experimentally measured values, as shown in Figure 4.

### 3.3. Effect of Variables on the ITZ Void Fraction

**Figure 5:**
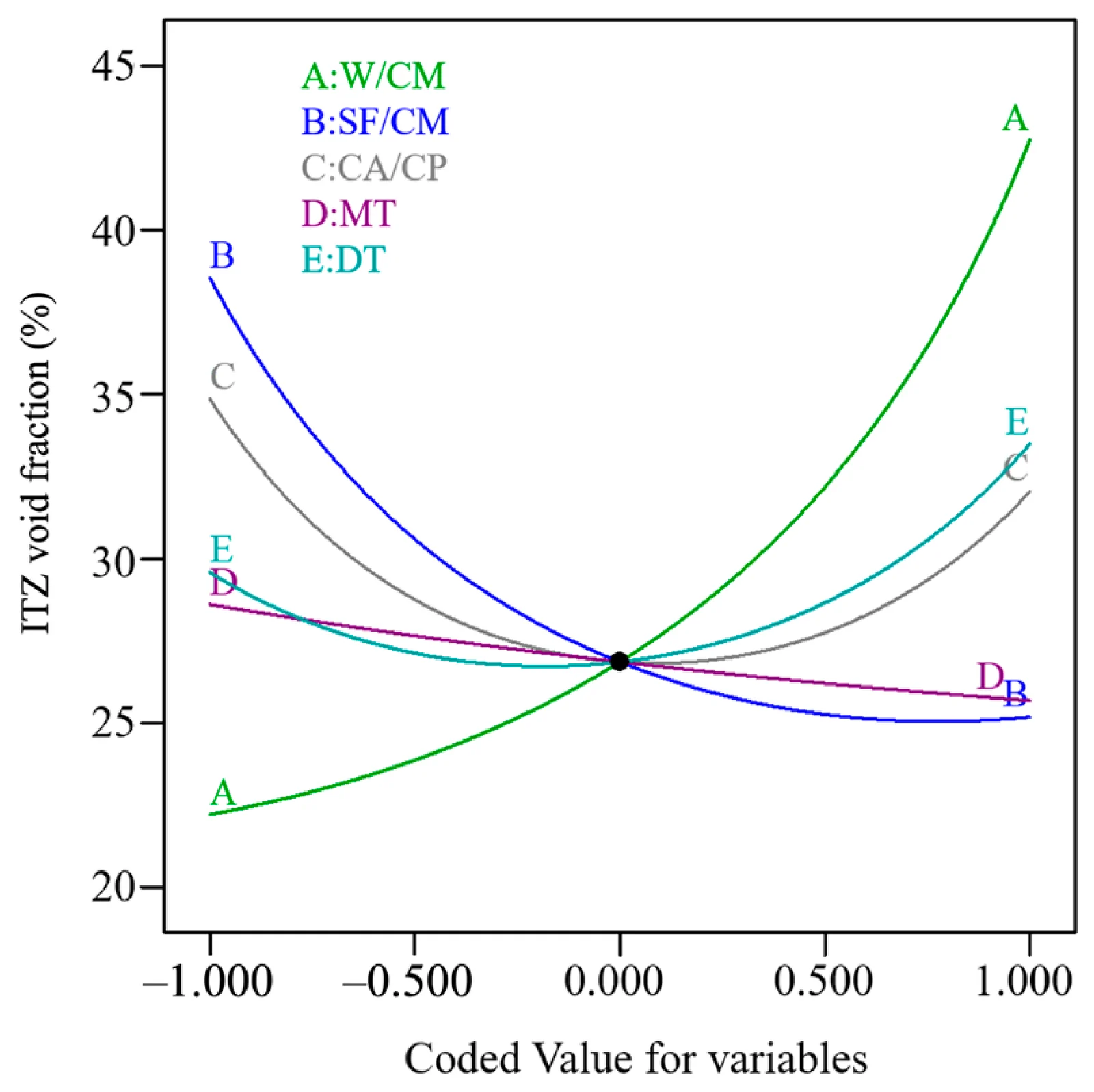
Effect of variables’ variation on the ITZ void fraction in HVFA concrete.

To illustrate the influence of different variables on the void fraction of the ITZ, perturbation plots are presented in Figure 5. The parameters of each variable are expressed in coded form, which facilitates a simplified comparison among the different variables. This study employed a five-factor experimental design, resulting in the coded values of the variables ranging from −1.82 to 1.82, covering five levels from the lowest to the highest [33]. For instance, the coded range of −1.82 to 1.82 for the variable SF/CM corresponds to actual values of 0 to 10%. In this case, the code values of −1 and 1 for SF/CM in Figure 3 represented 2.2% and 7.8% in actual value, respectively.

As illustrated in Figure 5, Equation (1), and Table 7, the variables W/CM, SF/CM, CA/CP, and MD exhibited a significant effect on the ITZ void fraction of HVFA concrete. While a low water-to-cementitious materials ratio generally reduced the porosity of the cement paste, an excessively low ratio can adversely affect both the pozzolanic reaction of silica fume and the hydration of cement clinker in the blended system. In fact, there exists a critical combination for the water-to-cementitious materials ratio and silica fume content, which ensures that the amounts of CH and moisture are sufficient for the pozzolanic reaction of silica fume [34], thereby densifying the microstructure of the ITZ.

Additionally, the mass ratio of coarse aggregate to cement paste also exhibited an optimal point in its influence on the ITZ void fraction of HVFA concrete. A lower CA/CP ratio resulted in a thicker cement paste coating on the coarse aggregate, providing a greater volume of coating material at the aggregate surface. This may enhance the interaction between the coating layer and the surrounding cementitious matrix during subsequent concrete preparation. In this case, it may dilute the local silica fume concentration in the ITZ of HVFA concrete, thereby reducing the effectiveness of the silica fume pozzolanic reaction in contributing to ITZ densification and reducing the ITZ void fraction. On the contrary, a higher CA/CP ratio may result in insufficient coverage of the coarse aggregate surface by the cement paste, which could similarly limit the effectiveness of the silica fume-modified coating in modifying the ITZ microstructure and reducing its void fraction. The potential formation of additional C-S-H gel through the pozzolanic reaction of silica fume may contribute to this densification effect.

Mixing time also had a significant effect on the ITZ void fraction. Within the investigated experimental range, increasing the mixing time generally decreased the ITZ void fraction, indicating that a longer mixing period was associated with a denser interfacial microstructure. This effect may be related to improved dispersion and distribution of the cementitious coating and its interaction with the surrounding matrix during the subsequent preparation of the concrete.

### 3.4. Interaction Between Variables

**Figure 6:**
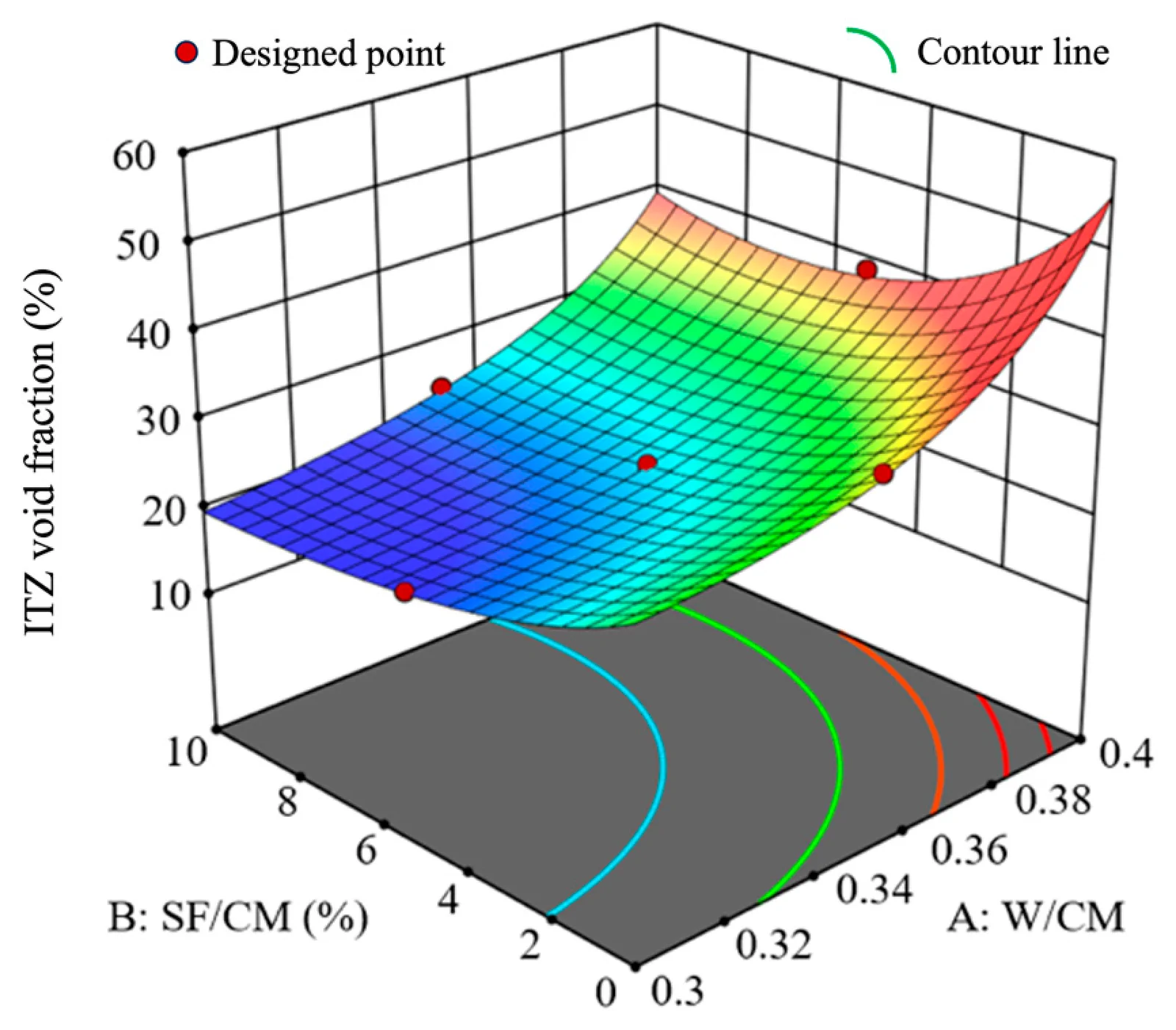
3D plot of the effect of W/CM and SF/CM on the ITZ void fraction.

In addition to the influence of aforementioned individual factors, interactions among the factors also existed for the model established in this study, as illustrated by the 3D response surface plots in Figure 6. This figure illustrates the interaction between two variables while keeping the other variables at their central levels. It can be seen from Figure 6 that the void fraction of the ITZ caused by the variation in W/CM was affected by changes in the level of SF/CM. In this case, there existed an optimal combination of W/CM and SF/CM that minimized the ITZ porosity of HVFA concrete [33]. As shown in Figure 6, when W/CM and SF/CM achieved their maximum value and minimum value, respectively, the ITZ void fraction of the ITZ increased significantly. Within this factor combination, adjusting any single variable directly affected the void fraction of the ITZ, and this impact was dependent on the other variable in the combination. The 3D response surface diagrams not only confirmed the individual influence of each variable on void fraction, but also clearly revealed their interdependencies, thereby providing a view for systematically optimizing the ITZ void fraction of HVFA concrete [35].

### 3.5. Optimization Solution and Its Verification

During the aforementioned process, the relationship between the variables and the response (ITZ void fraction) was elucidated. In order to obtain HVFA concrete with optimal performance, the optimization prediction was performed based on the response surface model. This study took the minimum void fraction of the ITZ in HVFA concrete as the optimization objective, while the optimization solution for all variables was set within defined ranges. Consequently, HVFA concrete with the optimal pretreatment of coarse aggregate with silica fume-modified cement paste can be obtained, as shown in Table 7.

It should be noted that drying time (*X*_5_) was included as one of the five factors in the response surface experimental design. However, its regression terms did not show a statistically significant contribution to ITZ void fraction within the investigated experimental range and were therefore not included in the reduced regression equation. Accordingly, the drying time should not be interpreted as a statistically significant factor governing the optimized ITZ void fraction. The value of 5 h reported for the selected pretreatment condition represents the drying condition associated with the selected experimental/optimization solution within the investigated range, rather than an independently established optimum drying time.

Based on the developed response surface model, the optimized combination of the investigated factors was determined within the experimental design domain. The optimized W/CM ratio was 0.3, which corresponded to the lower boundary of the investigated range, while the optimized SF/CM ratio of 9% and CA/CP ratio of 4 were close to the upper ends of their respective investigated ranges. Therefore, the obtained condition is considered the optimum within the investigated experimental domain rather than a general optimum. The proximity of several optimized factors to the boundaries also indicates that the actual optimum may lie outside the investigated design space. Further experiments extending the relevant factor ranges would be required to determine whether lower W/CM, higher SF/CM, or higher CA/CP values could produce further reductions in ITZ void fraction.

**Figure 7:**
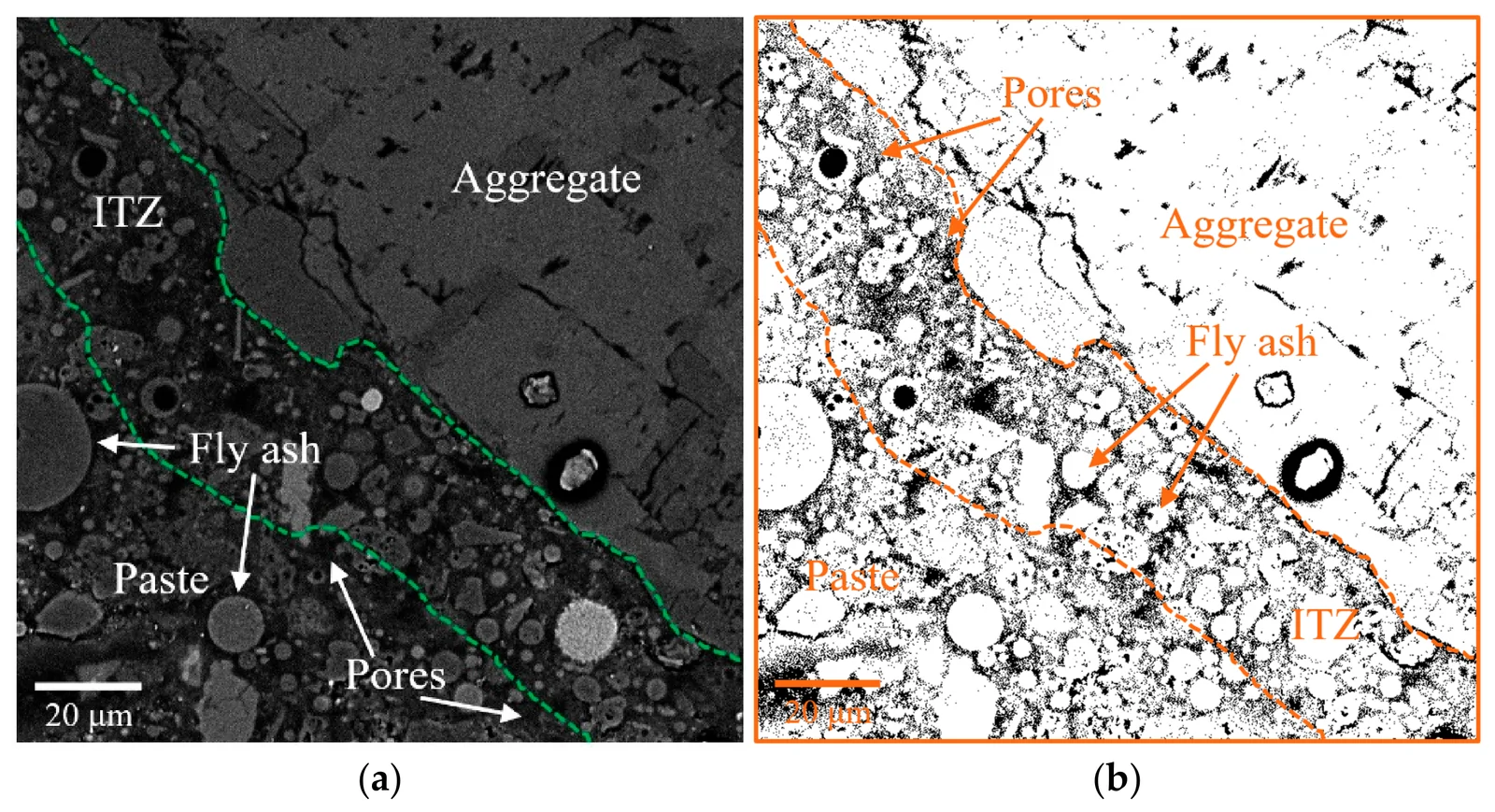
The microstructure of the ITZ in optimized HVFA concrete: (**a**) BSE image and (**b**) binary image.

Under the optimized condition, the model predicted a mean ITZ void fraction of 21.0%, with a standard deviation of 0.0134 and a 95% confidence interval for the predicted mean of 19.88–22.70%. Experimental confirmation was performed using one independently prepared specimen under the selected optimized condition, and the measured ITZ void fraction was 21.4%, as shown in Figure 7. As mentioned above, during this calculation, five aggregate–paste interfaces were selected from one specimen, and 10 randomly selected BSE images were acquired from each interface, resulting in 50 images of the optimized concrete. The ITZ void fraction was calculated for each image and then averaged to obtain the mean value for the concrete. Therefore, the reported ITZ void fraction of 21.4% for the experimentally confirmed optimized mixture represents the mean value obtained from the 50 analyzed BSE images. Because the 50 images were obtained from one specimen, they were treated as image-level subsamples rather than independent experimental replicates.

This measured result falls within the 95% confidence interval of the predicted mean and is close to the predicted value. This agreement between the predicted and measured values provides preliminary experimental confirmation of the selected optimized condition. However, because only one independently prepared specimen was used for confirmation, specimen-level variability could not be quantified. Therefore, the present result should be considered preliminary confirmation rather than comprehensive validation of the model. The assessment of the reproducibility of the predicted response using additional independently prepared specimens will be investigated in the future work.

### 3.6. Comparison of Optimized Concrete and Control Concrete

To further elucidate the performance enhancement effect of silica fume application in the ITZ of HVFA concrete, this study designed two control groups of HVFA concrete based on the factors of the optimal mix. As shown in Table 8, the first control group (Control 1) maintained all other variables unchanged while only altering the drying time, aiming to investigate the influence of drying time on the stability of the coating layer around coarse aggregate. In the second control group (Control 2), the raw coarse aggregate without any pretreatment was used, which allowed for evaluating the performance improvement of the optimal pretreatment compared to concrete using untreated coarse aggregate. With these three types of coarse aggregate, three groups of HVFA concrete were cast based on the mix proportions listed in Table 9.

It should be noted that the statistical significance of the pretreatment factors was evaluated only with respect to the ITZ void fraction in the response surface experiment. The subsequent comparison between the optimized concrete and Control 1 was conducted to compare the measured concrete properties under their respective preparation conditions and was not designed to isolate the independent effect of any individual pretreatment factors. Therefore, although differences in compressive strength and water transport behavior were observed between these two mixtures, these differences should not be interpreted as evidence of an independent effect of drying time or any other statistically nonsignificant factor in the RSM model.

#### 3.6.1. Compressive Strength

Table 10 presents the compressive strengths for the optimized and two control groups of HVFA concrete. The highest compressive strength was observed in the optimized HVFA concrete, reaching 40.2 MPa. This result indicates that pretreating coarse aggregate with silica fume-modified cement paste was associated with an improved ITZ microstructure and higher compressive strength. It is noteworthy that Control 1 exhibited a slightly lower compressive strength than the optimized concrete, with a value of 38.1 MPa. This difference suggests that the drying process during the pretreatment may contribute to the stabilization of the silica fume-modified cement paste within the ITZ, thereby densifying the ITZ microstructure and increasing the compressive strength of HVFA concrete. In contrast, Control 2, prepared with untreated coarse aggregates, exhibited a compressive strength of only 32.5 MPa, representing a 19.2% reduction compared with the optimized concrete.

Through the pretreatment approach, the coating layer applied to the coarse aggregate provided a localized region for silica fume to modify the aggregate–paste interface in HVFA concrete. The observed densification of the ITZ may be associated with the pozzolanic activity of silica fume. Previous studies have reported that the amorphous SiO_2_ in silica fume can react with CH released during cement hydration to form additional C-S-H gel, which may contribute to pore refinement and ITZ densification [36,37]. The denser ITZ may contribute to more favorable stress transfer between the aggregate and paste, which could partly explain the higher compressive strength observed for the optimized concrete.

It should be noted that only the 28-day compressive strength was measured in this study. Therefore, the age-dependent development of strength and the respective contributions of silica fume and fly ash reactions cannot be directly evaluated. Further investigations involving strength measurements at multiple curing ages and corresponding microstructural analyses are needed to clarify these mechanisms.

#### 3.6.2. Water Sorptivity

**Figure 8:**
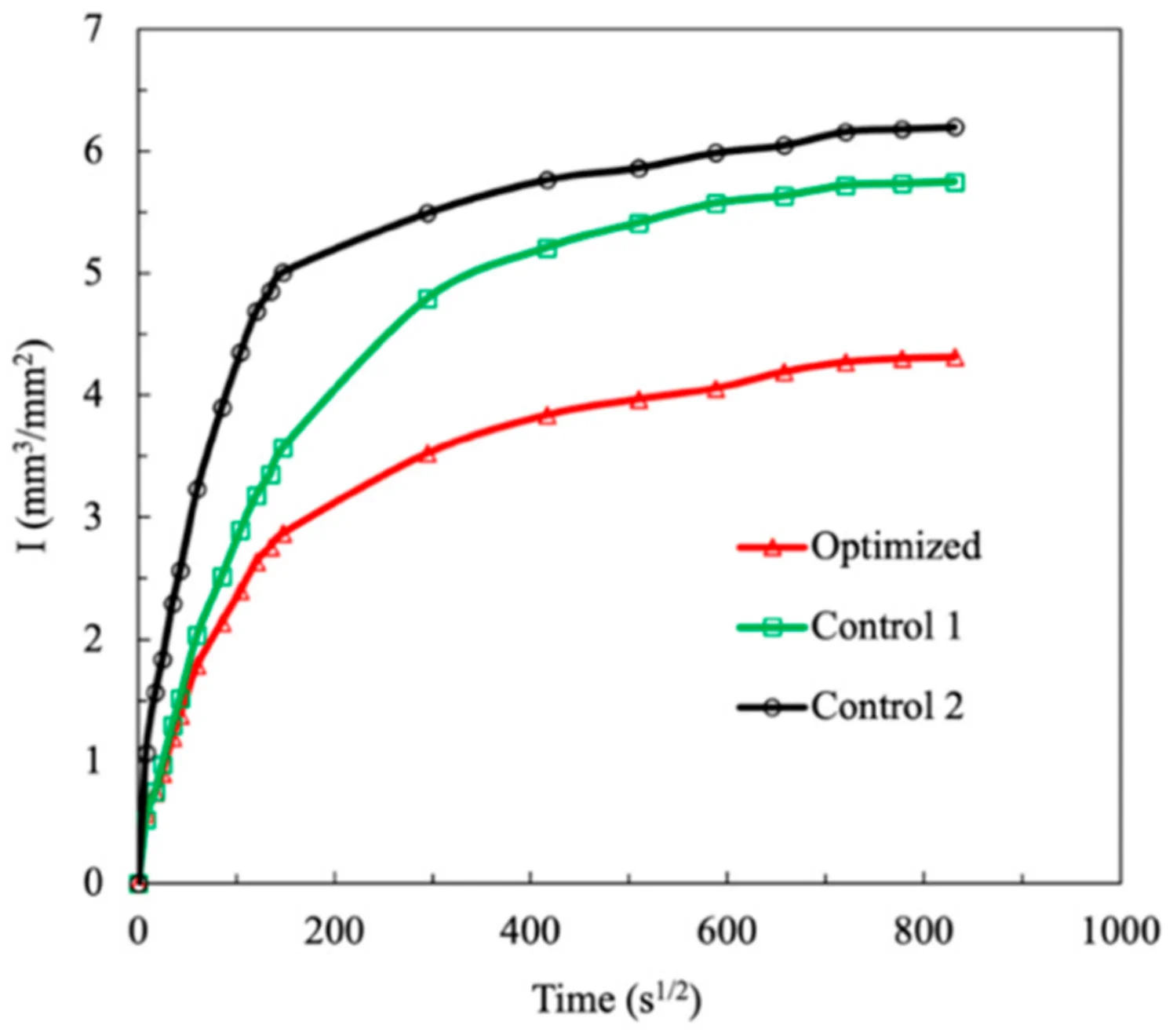
Water absorption curves for HVFA concretes.

The water absorption test was conducted in accordance with ASTM C1585. Only the bottom surface of the concrete was exposed to water, and the variation of the amount of water absorption normalized by the surface area of concrete over time is shown in Figure 8. It can be observed that the optimized concrete exhibited the lowest amount of water absorption at the end of the test. This can be attributed to the improved ITZ in the optimized concrete, where the denser microstructure hindered moisture transport, thereby reducing water absorption. Moreover, observation reveals that as time progressed, the water absorption increased rapidly in the early stage and slowly in the later stage, indicating that the absorption process can be divided into two distinct phases. Linear fitting of the data points from the first 6 h and from 1 to 8 days yields the initial and secondary water sorptivity, respectively. The sorptivity coefficient, represented by the slope of the fitted curve, reflects the rate of water absorption of HVFA concrete, as listed in Table 11.

The initial sorptivity coefficient is typically much greater than the secondary sorptivity coefficient, as the former represents the water absorption rate through larger pores in concrete [38]. As shown in Table 11, the optimized concrete exhibited the lowest initial sorptivity coefficient, at only 0.0181 mm/s^1/2^, representing reductions of 22.0% and 41.4% compared with those of Control 1 and Control 2, respectively. This suggests that the presence of silica fume in the ITZ may have contributed to pore structure refinement through its pozzolanic activity, which may have influenced the moisture transport behavior of the concrete.

The secondary sorptivity coefficient reflects the rate of water uptake during the later stage of the sorptivity test. As shown in Table 11, the optimized concrete exhibited a higher secondary sorptivity coefficient than concrete Control 2. This suggests that the optimized concrete had a higher rate of water uptake during the later stage of the test. Such behavior may be related to differences in the pore structure and moisture transport characteristics of the concrete, including those of the ITZ. In addition, since the pore structure of the ITZ may influence the transport and redistribution of water during freeze-thaw exposure, the potential relationship between the modified ITZ, water transport behavior, and freeze-thaw resistance warrants further investigation.

#### 3.6.3. Microstructure Investigation

**Figure 9:**
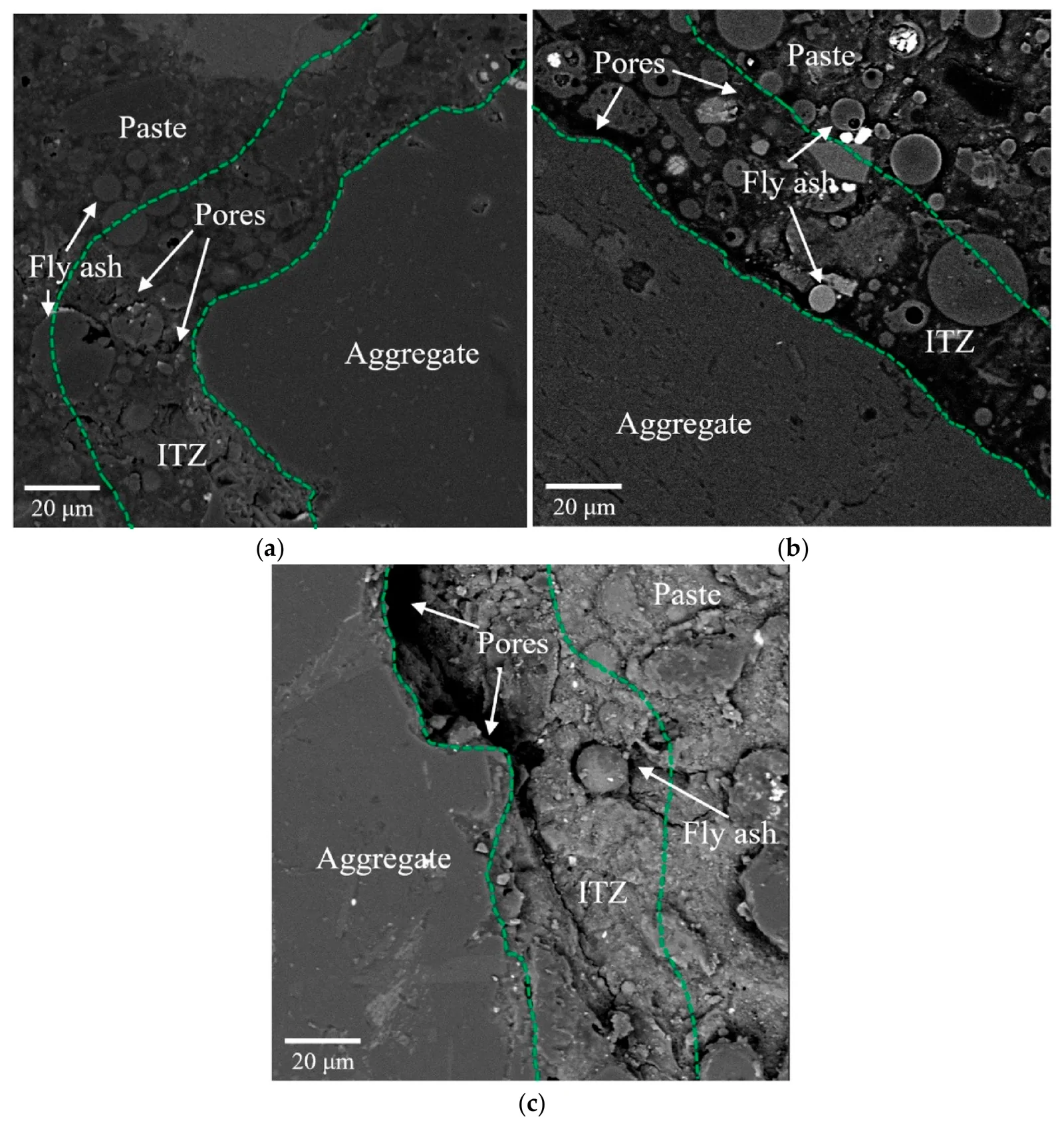
BSE image of the ITZ for (**a**) Optimized, (**b**) Control 1, and (**c**) Control 2 HVFA concretes.

Figure 9 presents the BSE images of the three groups of HVFA concrete to reveal the microstructural differences in the ITZ regions. Evidently, the BSE image of the optimized HVFA concrete exhibited a denser and more uniform ITZ microstructure, indicating the positive effect of the pretreatment of coarse aggregate, which is likely beneficial for enhancing the mechanical properties and impermeability of HVFA concrete. In contrast, concrete Control 1 showed a less uniform ITZ microstructure, which may be attributed to the lack of a drying step during the pretreatment of the coarse aggregate, thereby compromising the integrity of the ITZ. Moreover, the ITZ of concrete Control 2, which was prepared using untreated coarse aggregate, exhibited a significantly more irregular and non-uniform morphology. This can be attributed to the absence of ITZ modification, which resulted in higher void fraction and a less densified interfacial microstructure.

**Figure 10:**
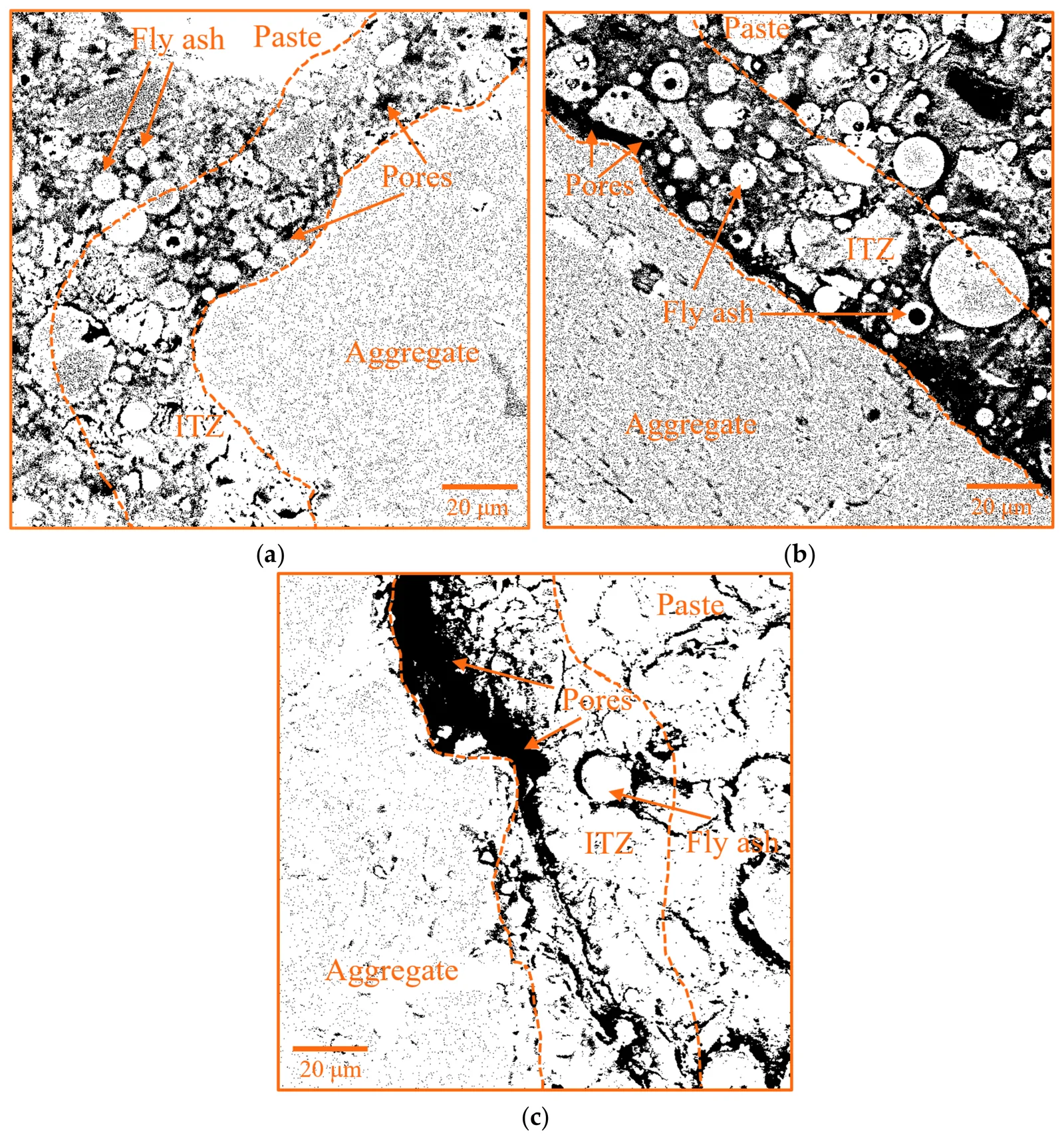
Segmentation of pores as black pixels in binary image for (**a**) Optimized, (**b**) Control 1, and (**c**) Control 2 HVFA concretes.

To quantify the ITZ void fraction of three groups of HVFA concrete, the pores and cracks in the ITZ region were segmented as black pixels in the binarized images, as shown in Figure 10. The results of the ITZ void fraction in these concretes are provided in Table 12. Note that the reported standard deviation was calculated from the five interface-level mean values. Clearly, the optimized concrete incorporating pretreated coarse aggregate exhibited the lowest void fraction of 21.4%, indicating a denser ITZ region. Concrete Control 1 showed an ITZ void fraction of 33.0%, with a relatively looser structure, which confirms the importance of the drying process during the pretreatment of coarse aggregate. Concrete Control 2, prepared with untreated coarse aggregates, had the highest void fraction, reaching 44.0%, further demonstrating the effectiveness of the approach proposed in this study in improving the ITZ.

### 3.7. Material-Use Advantages

In concrete, the dosage of silica fume is an important factor affecting the microstructure of the ITZ because the fine particles and pozzolanic reaction of silica fume can contribute to ITZ densification. Previous studies have reported that silica fume replacement levels of approximately 5–15% can improve the mechanical properties and durability of concrete, with levels of 8–10% commonly adopted [39,40,41,42]. To provide a consistent assessment of the overall silica fume consumption, the total binder was defined as the sum of all cementitious materials used in both the bulk matrix and the coating paste. As listed in Table 10, the total cementitious material for the optimized mixture consisted of 160 g of cement and 240 g of fly ash in the bulk matrix, together with 175 g of cement and 17 g of silica fume in the coating paste. Therefore, the total cementitious material was 592 g, and the overall silica fume dosage was calculated as 17/592 = 2.87%, approximately 2.9 wt.%. Compared with the commonly reported silica fume replacement levels of 8–10%, the overall silica fume dosage of 2.9% in the present study was approximately 2.8–3.5 times lower, corresponding to a reduction of 64–71%.

Despite the substantially lower overall silica fume dosage, the localized pretreatment strategy effectively modified the ITZ and improved the mechanical and water transport-related properties of HVFA concrete. The reduced silica fume consumption demonstrates a potential material-use advantage of the proposed pretreatment strategy. However, this comparison with conventional silica fume dosages reported in the literature does not by itself demonstrate that targeted silica fume application is more efficient than conventional direct mixing. A direct-mixing control with an equivalent silica fume dosage and otherwise comparable mixture composition would be required to determine whether localized application provides superior ITZ modification or concrete performance per unit mass of silica fume in future work.

Furthermore, compared to concrete Control 1, the optimized concrete offered a 5.5% increase in compressive strength and a 16.73% reduction in the initial sorptivity coefficient. Through the application of the silica fume-modified cement paste coating on the coarse aggregate and the subsequent drying process, the optimized concrete fully utilized the high reactivity of silica fume.

## 4. Discussion

Although the aggregate pretreatment approach was demonstrated at the laboratory scale, its practical implementation in ready-mix or precast concrete production requires consideration of coating uniformity, processing time, and drying conditions. At a larger scale, the silica fume-modified cement paste could potentially be applied to coarse aggregates using continuous or batch-type mixing equipment specifically designed to promote uniform paste distribution around the aggregate surfaces. Control of the aggregate-to-paste ratio, mixing time, and mixing intensity would be important for maintaining a relatively uniform coating thickness. In addition, coating uniformity could be monitored through periodic sampling and image-based inspection of representative coated aggregates. Quantitative measurements of coating thickness at multiple locations would provide a more rigorous evaluation of coating uniformity and will be considered in future work.

The drying step is another practical consideration. The several-hour drying period used in the present laboratory procedure was intended to stabilize the coating before concrete mixing and should not be directly regarded as an industrially optimized processing time. In precast production, the coated aggregates could potentially be prepared in advance and stored before concrete casting, making the drying step less restrictive. For ready-mix production, however, a prolonged drying period may reduce production efficiency and would require optimization. Accelerated drying under controlled temperature and humidity, or adjustment of the coating formulation to achieve sufficient coating stability with a shorter conditioning period, may provide possible approaches for reducing processing time.

A direct comparison with conventional silica fume addition to the bulk HVFA concrete would provide further insight into the efficiency of the proposed localized silica fume utilization strategy. In the present study, however, silica fume was intentionally introduced through the coarse-aggregate coating rather than directly incorporated into the bulk matrix, and a corresponding control mixture with silica fume directly mixed into the HVFA concrete was not included in the experimental design. Nevertheless, the optimized pretreatment required only 17 g of silica fume, corresponding to approximately 2.9 wt.% of the total cementitious materials used in the bulk matrix and coating paste. This indicates that a relatively small amount of silica fume can be concentrated at the aggregate–paste interface, where ITZ modification was the primary objective of the pretreatment. In contrast, direct addition of silica fume to the bulk matrix distributes the material throughout the concrete and may therefore require a higher overall silica fume dosage to achieve a comparable modification of the ITZ. However, the relative efficiency of these two utilization strategies depends on their effects on workability, strength, transport properties, processing requirements, and silica fume consumption and cannot be determined from the present dataset alone.

It should be noted that the optimized concrete and Control 2 differed in their mixture compositions because the aggregate pretreatment process introduced additional cementitious materials and water through the coating paste. Therefore, the optimized concrete contained a greater total amount of binder and paste than Control 2. The differences in compressive strength and water transport behavior between these two mixtures may consequently reflect the combined effects of the modified ITZ and the difference in overall mixture composition. To address this limitation, Control 1, which was prepared using the same overall material quantities as the optimized concrete but without the drying step, provides a more appropriate comparison for evaluating the effect of the aggregate pretreatment process under an equivalent overall mixture composition. The comparison between the optimized concrete and Control 1, therefore, provides evidence regarding the influence of the drying/conditioning stage while maintaining comparable overall material contents. A rigorously matched untreated aggregate control featuring the same total amount of binder will be considered in future work. Additional controls, including untreated aggregate with composition-matched additional paste, cement paste-coated aggregate without silica fume, and direct mixing of silica fume at equivalent and conventional dosages, would be required to isolate the effects of additional paste, coating, silica fume, drying, and targeted silica fume placement. These comparisons should also be addressed in future work.

## 5. Conclusions

This study proposed an aggregate pretreatment approach to improve the ITZ in HVFA concrete by coating the coarse aggregate using a silica fume-modified cement paste. The effectiveness of this approach in enhancing the ITZ was evaluated based on porosity obtained through image analysis, and the main conclusions are as follows:

(1) Among the five investigated pretreatment factors, the W/CM, SF/CM, and CA/CP ratios, as well as mixing time (MT), had significant effects on the ITZ void fraction within the investigated experimental domain. For these factors, the ITZ void fraction initially decreased and then increased with increasing factor levels, indicating the existence of favorable parameter ranges for ITZ modification.
(2) An interaction between W/CM and SF/CM was observed in their effects on the ITZ void fraction of HVFA concrete. Specifically, a critical combination of W/CM and SF/CM was identified for reducing the ITZ void fraction.
(3) Aiming to minimize the ITZ void fraction, the response surface optimization identified W/CM = 0.30, SF/CM = 9%, CA/CP = 4, and MT = 3 min as the factor setting within the investigated experimental domain. Since DT was not statistically significant in the final model, a drying time of 5 h was selected as the processing condition for experimental confirmation. Coarse aggregate prepared with these factors were then used to produce the optimized HVFA concrete, and the resulting ITZ void fraction was found to be only 21.4%. The close agreement between the predicted and measured values provided preliminary experimental confirmation of the optimization result.
(4) Compared with the control groups of HVFA concrete, the optimized HVFA concrete demonstrated the highest compressive strength and the lowest ITZ void fraction. It also showed a lower initial sorptivity coefficient and a higher secondary sorptivity coefficient than Control 2, indicating a lower and a higher rate of water absorption during the early and later stage of water absorption, respectively.
(5) This approach employed a silica fume dosage that was 2.8–3.5 times lower than commonly reported dosages while still achieving favorable mechanical properties and durability, indicating its potential material-use advantages.

## Figures and Tables

**Table 1:** Chemical composition of cement, silica fume, and fly ash (% by weight).

Chemical Composition	Cement	Silica Fume	Fly Ash
Al_2_O_3_	4.83	0.58	32.44
SiO_2_	20.50	93.00	49.20
Fe_2_O_3_	3.24	2.79	4.76
CaO	63.20	0.60	7.47
MgO	3.22	1.00	0.81
Na_2_O	0.15	1.00	0.51
K_2_O	1.29	0.10	1.82
SO_3_	2.66	0.50	0.55
LOI *	0.91	0.43	1.44

* Loss on ignition.

**Table 2:** Physical properties of cement, silica fume, and fly ash.

Property	Cement	Silica Fume	Fly Ash
Specific gravity	3.10	2.20	2.10
Surface area (m^2^/kg)	340	1500	520
Average particle size (μm)	15.23	0.20	20.46

**Table 3:** Actual value for the factors.

Factor	Value				
	−α	−1	0	+1	+α
W/CM (*X*_1_)	0.3	0.32	0.35	0.38	0.4
SF/CM (*X*_2_)	0	2.2%	5%	7.8%	10%
CA/CP (*X*_3_)	1.5	2.2	3	3.8	4.5
MT (*X*_4_)	2 min	3.8 min	6 min	8.2 min	10 min
DT (*X*_5_)	0	1.8 h	4 h	6.2 h	8 h

**Table 4:** Variables and levels selected for the preparation of coated coarse aggregate.

Mix	*X*_1_ W/CM	*X*_2_ SF/CM (%)	*X*_3_ CA/CP	*X*_4_ MT (min)	*X*_5_ DT (h)
1	0.38	7.8	2.2	8.2	1.8
2	0.35	5	3	10	4
3	0.35	5	3	6	4
4	0.35	5	3	6	4
5	0.35	5	4.5	6	4
6	0.35	5	3	6	4
7	0.38	7.8	2.2	3.8	6.2
8	0.35	5	3	6	8
9	0.3	5	3	6	4
10	0.38	7.8	3.8	3.8	1.8
11	0.35	5	3	6	4
12	0.32	2.2	3.8	8.2	6.2
13	0.38	2.2	3.8	3.8	6.2
14	0.32	7.8	3.8	3.8	6.2
15	0.32	7.8	3.8	8.2	1.8
16	0.35	5	3	2	4
17	0.38	2.2	2.2	8.2	6.2
18	0.35	5	3	6	0
19	0.32	2.2	2.2	3.8	1.8
20	0.35	5	3	6	4
21	0.38	2.2	3.8	8.2	1.8
22	0.35	5	1.5	6	4
23	0.35	0	3	6	4
24	0.4	5	3	6	4
25	0.32	7.8	2.2	8.2	6.2
26	0.35	10	3	6	4

**Table 5:** The void fraction results of ITZ in concrete (mean ± SD).

**Mix**	**1**	**2**	**3**	**4**	**5**	**6**	**7**	**8**	**9**	**10**
Void fraction (%)	34.6 ± 2.6	24.8 ± 1.9	25.0 ± 2.1	25.3 ± 1.8	30.0 ± 2.4	24.8 ± 1.7	33.7 ± 2.8	31.2 ± 2.3	22.0 ± 1.6	33.1 ± 2.5
**Mix**	**11**	**12**	**13**	**14**	**15**	**16**	**17**	**18**	**19**	**20**
Void fraction (%)	25.5 ± 2.0	27.6 ± 2.2	37.5 ± 3.1	23.7 ± 1.8	23.4 ± 1.7	27.2 ± 2.1	38.4 ± 3.2	28.0 ± 2.3	27.8 ± 2.0	25.7 ± 1.9
**Mix**	**21**	**22**	**23**	**24**	**25**	**26**	**-**	**-**	**-**	**-**
Void fraction (%)	37.2 ± 3.0	32.3 ± 2.5	35.4 ± 2.8	39.0 ± 3.3	23.5 ± 1.8	24.4 ± 1.9	-	-	-	-

**Table 6:** ANOVA results for the proposed regression model.

Source	Coefficient	Sum of Squares	Degree of Freedom	Mean Square	F-Value	*p*-Value
Model	0.0158, intercept	0.0002	20	0.0000	43.85	0.0003
X_1_-W/C	−0.0047	0.0000	1	0.0000	190.34	0.0001
X_2_-S/CM	0.0025	0.0000	1	0.0000	77.71	0.0003
X_3_-CA/CP	0.0003	6.732 × 10^−7^	1	6.732 × 10^−7^	11.44	0.0196
X_4_-MT	−0.0003	1.674 × 10^−6^	1	1.674 × 10^−6^	7.46	0.0412
X_1_X_2_	−0.0015	8.363 × 10^−7^	1	8.363 × 10^−7^	16.00	0.0103
X_1_^2^	−0.0014	4.150 × 10^−6^	1	4.150 × 10^−6^	16.00	0.0103
X_2_^2^	−0.0020	7.989 × 10^−6^	1	7.989 × 10^−6^	30.80	0.0026
X_3_^2^	−0.0034	0.0000	1	0.0000	80.69	0.0003
X_4_^2^	−0.0025	3.454 × 10^−8^	1	3.454 × 10^−8^	45.20	0.0011
Residual	-	1.297 × 10^−6^	5	2.594 × 10^−7^	-	-
Lack of Fit	-	9.447 × 10^−7^	1	9.447 × 10^−7^	0.1073	0.7306
Pure Error	-	3.522 × 10^−7^	4	8.804 × 10^−8^	-	-

**Table 7:** Goal for response and corresponding solution for HVFA concrete.

Response	Goal	Solution					Predicted Value
		*X*_1_	*X*_2_	*X*_3_	*X*_4_	*X*_5_ *	
ITZ void fraction	Minimize	0.3	9%	4	3 min	5 h	21.0%

* Note that drying time (*X*_5_) was not statistically significant, and 5 h was selected as the processing condition for the confirmation mixture.

**Table 8:** Pretreatment of coarse aggregate in three groups.

Group	W/CM	SF/CM	CA/CP	MT	DT
Optimized	0.3	9%	4	3 min	5 h
Control 1	0.3	9%	4	3 min	0
Control 2	No treatment				

**Table 9:** Mix design for three groups of HVFA concrete.

Component	Optimized	Control 1	Control 2
Cement in bulk matrix	160 kg/m^3^	160 kg/m^3^	160 kg/m^3^
Fly ash in bulk matrix	240 kg/m^3^	240 kg/m^3^	240 kg/m^3^
Water in bulk matrix	120 kg/m^3^	120 kg/m^3^	120 kg/m^3^
Sand	900 kg/m^3^	900 kg/m^3^	900 kg/m^3^
Coarse aggregate	Pretreated, 1000 kg/m^3^	No drying, 1000 kg/m^3^	Raw, 1000 kg/m^3^
Cement in coating	175 kg/(1000 kg CA)	175 kg/(1000 kg CA)	–
Silica fume in coating	17 kg/(1000 kg CA)	17 kg/(1000 kg CA)	–
Water in coating	58 kg/(1000 kg CA)	58 kg/(1000 kg CA)	–

Note: Bulk matrix proportions are expressed per cubic meter of concrete mixture. Coating paste constituents are expressed per 1000 kg of uncoated SSD coarse aggregate used for pretreatment.

**Table 10:** Compressive strength of HVFA concrete in three groups (mean ± SD).

Group	Optimized	Control 1	Control 2
Compressive strength (MPa)	40.2 ± 1.2	38.1 ± 1.1	32.5 ± 1.0

**Table 11:** Water sorptivity coefficient of HVFA concrete in three groups (mean ± SD).

Group	Initial Coefficient (mm/s^1/2^)	R^2^	Secondary Coefficient (mm/s^1/2^)	R^2^
Optimized	0.0181 ± 0.0005	0.9597	0.0015 ± 0.0002	0.9725
Control 1	0.0232 ± 0.0006	0.9778	0.0017 ± 0.0002	0.9542
Control 2	0.0309 ± 0.0008	0.9420	0.0013 ± 0.0002	0.9639

**Table 12:** ITZ void fraction results in three groups of HVFA concretes (mean ± SD).

Group	Optimized	Control 1	Control 2
ITZ void fraction (%)	21.4 ± 1.6	33.0 ± 2.5	44.0 ± 3.5

## Data Availability

The original contributions presented in this study are included in the article. Further inquiries can be directed to the corresponding authors.

## References

[1] Shuang N., Chen B., Lu X., Tian B., Xiong B., Zhang J. (2025). A Chemo-Transport-Damage Mesoscopic Model Quantifying the Influence of ITZ for Concrete under Sulfate Attack. Constr. Build. Mater..

[2] Hu H., Ma X., Zuo J., Wei Y., She A., Yao W., Wu M. (2026). Improvement of Concrete Performance through a Ternary Aggregate System: Microstructural Insights into Pore Structure and ITZ Characteristics. Cem. Concr. Compos..

[3] Guan X., Ji H., Qiu J., Zhang Y., Yu J., Li L. (2024). Chloride Ion Permeability of Concrete Containing Recycled Composite Powder from Building Demolition Waste. Constr. Build. Mater..

[4] Chen Y., Chen M., Chen R., Kang X. (2023). Stray Current Induced ITZ Effect on Chloride Transport in Concrete. Constr. Build. Mater..

[5] Tang Q., Chai S., Hou D., Yin B., Wang M., Liu P., Hu H., Zhao X., Wang P. (2024). Unraveling Concrete’s Interfacial Transition Zone Vulnerability under Erosive Environments: A Molecular Dynamics Study. J. Build. Eng..

[6] Xiao T., Liang X., Du S. (2024). Development of High-Volume Fly Ash Concrete with Improved Interfacial Transition Zone. J. Build. Eng..

[7] Herath C., Law D.W., Gunasekara C., Setunge S. (2023). Sulphate and Acid Resistance of HVFA Concrete Incorporating Nano Silica. Constr. Build. Mater..

[8] Torii K., Kawamura M. (1994). Effects of Fly Ash and Silica Fume on the Resistance of Mortar to Sulfuric Acid and Sulfate Attack. Cem. Concr. Res..

[9] Shaikh F.U.A., Supit S.W.M. (2015). Compressive Strength and Durability Properties of High Volume Fly Ash (HVFA) Concretes Containing Ultrafine Fly Ash (UFFA). Constr. Build. Mater..

[10] Haha M.B., De Weerdt K., Lothenbach B. (2010). Quantification of the Degree of Reaction of Fly Ash. Cem. Concr. Res..

[11] Du S., Zhao Q., Shi X. (2023). Quantification of the Reaction Degree of Fly Ash in Blended Cement Systems. Cem. Concr. Res..

[12] Li Y., Wang R., Li S., Zhao Y., Qin Y. (2018). Resistance of Recycled Aggregate Concrete Containing Low- and High-Volume Fly Ash against the Combined Action of Freeze–Thaw Cycles and Sulfate Attack. Constr. Build. Mater..

[13] Kuroda M., Watanabe T., Terashi N. (2000). Increase of Bond Strength at Interfacial Transition Zone by the Use of Fly Ash. Cem. Concr. Res..

[14] Gao J.M., Qian C.X., Liu H.F., Wang B., Li L. (2005). ITZ Microstructure of Concrete Containing GGBS. Cem. Concr. Res..

[15] Chen Y., Du S. (2025). Improving the Interfacial Transition Zone of Concrete: A Targeted Approach Utilizing Graphene Oxide. Constr. Build. Mater..

[16] Chen Y., Zhou K., Du S. (2025). Introducing Carbon Nanotubes into Concrete: A Porosity-Based Characterization of Dispersion. Constr. Build. Mater..

[17] Amjad H., Ahmad F., Irshad Qureshi M. (2023). Enhanced Mechanical and Durability Resilience of Plastic Aggregate Concrete Modified with Nano-Iron Oxide and Sisal Fiber Reinforcement. Constr. Build. Mater..

[18] Li L., Poon C.S., Xiao J., Xuan D. (2017). Effect of Carbonated Recycled Coarse Aggregate on the Dynamic Compressive Behavior of Recycled Aggregate Concrete. Constr. Build. Mater..

[19] Peng L., Zeng W., Zhao Y., Li L., Poon C., Zheng H. (2022). Steel Corrosion and Corrosion-Induced Cracking in Reinforced Concrete with Carbonated Recycled Aggregate. Cem. Concr. Compos..

[20] Zhao Y., Peng L., Zeng W., Poon C.S., Lu Z. (2021). Improvement in Properties of Concrete with Modified RCA by Microbial Induced Carbonate Precipitation. Cem. Concr. Compos..

[21] García-González J., Rodríguez-Robles D., Wang J., De Belie N., Morán-del Pozo J.M., Guerra-Romero M.I., Juan-Valdés A. (2017). Quality Improvement of Mixed and Ceramic Recycled Aggregates by Biodeposition of Calcium Carbonate. Constr. Build. Mater..

[22] Wu C.-R., Hong Z.-Q., Zhang J.-L., Kou S.-C. (2020). Pore Size Distribution and ITZ Performance of Mortars Prepared with Different Bio-Deposition Approaches for the Treatment of Recycled Concrete Aggregate. Cem. Concr. Compos..

[23] Lu D., Wang D., Wang Y., Zhong J. (2023). Nano-Engineering the Interfacial Transition Zone between Recycled Concrete Aggregates and Fresh Paste with Graphene Oxide. Constr. Build. Mater..

[24] Lyu K., Chen X., Liu X., Xie X., Shi J., Xia Q., Shah S.P. (2025). A Comparative Study of Utilizing Silica Powder and Nano-Based Silica to Enhance the Properties of MSWIFA-Cement Based Composites. Constr. Build. Mater..

[25] Du S., Ge X., Zhao Q. (2022). Central Composite Design-Based Development of Eco-Efficient High-Volume Fly Ash Mortar. Constr. Build. Mater..

[26] (2019). Standard for Test Methods of Concrete Physical and Mechanical Properties.

[27] (2020). Standard Test Methods for Measurement of Rate of Absorption of Water by Hydraulic-Cement Concretes.

[28] Du S., Shi X., Ge Y. (2017). Electron Probe Microanalysis Investigation into High-Volume Fly Ash Mortars. J. Mater. Civ. Eng..

[29] Wong H.S., Head M.K., Buenfeld N.R. (2006). Pore Segmentation of Cement-Based Materials from Backscattered Electron Images. Cem. Concr. Res..

[30] Li L., Yang J., Shen X. (2022). Measuring the Hydration Product Proportion in Composite Cement Paste by Using Quantitative BSE-EDS Image Analysis: A Comparative Study. Measurement.

[31] Fallah-Valukolaee S., Mousavi R., Arjomandi A., Nematzadeh M., Kazemi M. (2022). A Comparative Study of Mechanical Properties and Life Cycle Assessment of High-Strength Concrete Containing Silica Fume and Nanosilica as a Partial Cement Replacement. Structures.

[32] Abdulkadir I., Mohammed B.S., Liew M.S., Wahab M.M.A. (2021). Modelling and Optimization of the Mechanical Properties of Engineered Cementitious Composite Containing Crumb Rubber Pretreated with Graphene Oxide Using Response Surface Methodology. Constr. Build. Mater..

[33] Ghoddousi P., Adelzade Saadabadi L. (2022). Effect of Pore Physical and Chemical Microstructure Properties on Durability and Rebar Corrosion of Self-Compacting Concretes Containing Silica Fume and Metakaolin. J. Mater. Civ. Eng..

[34] Chu S.H., Kwan A.K.H. (2019). Co-Addition of Metakaolin and Silica Fume in Mortar: Effects and Advantages. Constr. Build. Mater..

[35] Kumar S., Kumar D.R., Wipulanusat W., Keawsawasvong S. (2024). Development of ANN-Based Metaheuristic Models for the Study of the Durability Characteristics of High-Volume Fly Ash Self-Compacting Concrete with Silica Fume. J. Build. Eng..

[36] Yavuz D., Akbulut Z.F., Guler S. (2024). Porous Concrete Modification with Silica Fume and Ground Granulated Blast Furnace Slag: Hydraulic and Mechanical Properties before and after Freeze-Thaw Exposure. Constr. Build. Mater..

[37] Mizan M.H., Shakik A., Matsumoto K. (2026). Effectiveness of Silica Fume on the Damage Behavior under Freeze-Thaw Cycles of the Concrete-PCM Interface. J. Build. Eng..

[38] Zhang Z., Angst U. (2020). A Dual-Permeability Approach to Study Anomalous Moisture Transport Properties of Cement-Based Materials. Transp. Porous Media.

[39] Wang H., Zhu P., Yan X., Liu H., Zhu L., Wang X. (2023). Effect of Silica Fume on Frost Resistance and Recyclability Potential of Recycled Aggregate Concrete under Freeze–Thaw Environment. Constr. Build. Mater..

[40] Borosnyói A. (2016). Long Term Durability Performance and Mechanical Properties of High Performance Concretes with Combined Use of Supplementary Cementing Materials. Constr. Build. Mater..

[41] Bai W., Song Z., Yuan C., Guan J., Xie C., Huang H., Ma Y. (2023). Study on Mechanical Properties and Damage Mechanism of Recycled Concrete Containing Silica Fume in Freeze–Thaw Environment. Constr. Build. Mater..

[42] Wang D., Zhou X., Meng Y., Chen Z. (2017). Durability of Concrete Containing Fly Ash and Silica Fume against Combined Freezing-Thawing and Sulfate Attack. Constr. Build. Mater..

